# Anxiety increases information-seeking in response to large changes

**DOI:** 10.1038/s41598-022-10813-9

**Published:** 2022-05-05

**Authors:** Caroline J. Charpentier, Irene Cogliati Dezza, Valentina Vellani, Laura K. Globig, Maria Gädeke, Tali Sharot

**Affiliations:** 1 Division of Humanities and Social Sciences, California Institute of Technology, Pasadena, CA USA; 2 Institute of Cognitive Neuroscience, University College London, London, UK; 3 Department of Experimental Psychology, University College London, London, WC1H 0AP UK; 4 The Max Planck UCL Centre for Computational Psychiatry and Ageing Research, University College London, London, WC1B 5EH UK; 5 Department of Experimental Psychology, Ghent University, 9000 Ghent, Belgium; 6 Division of Medical Psychology, University of Bonn, 53113 Bonn, Germany; 7 Department of Brain and Cognitive Sciences, Massachusetts Institute of Technology, Cambridge, MA USA

**Keywords:** Emotion, Human behaviour

## Abstract

Seeking information when anxious may help reduce the aversive feeling of uncertainty and guide decision-making. If information is negative or confusing, however, this may increase anxiety further. Information gathered under anxiety can thus be beneficial and/or damaging. Here, we examine whether anxiety leads to a general increase in information-seeking, or rather to changes in the type of information and/or situations in which it is sought. In two controlled laboratory studies, we show that both trait anxiety and induced anxiety lead to a selective alteration in information-seeking. In particular, anxiety did not enhance the general tendency to seek information, nor did it alter the valence of the information gathered. Rather, anxiety amplified the tendency to seek information more in response to large changes in the environment. This was true even when the cause of the anxiety was not directly related to the information sought. As anxious individuals have been shown to have problems learning in changing environments, greater information-seeking in such environments may be an adaptive compensatory mechanism.

## Introduction

Gathering information can be an adaptive response to feeling anxious. This is because information can increase one’s sense of control, reduce an aversive sense of uncertainty, and help guide actions^1–3^. However, if the information revealed is negative or confusing, it can further increase anxiety^4–7^. Thus, the information people seek out when anxious could have a beneficial or a damaging effect on their well-being. Here, we examine whether and how anxiety alters the features of information people seek out.

While anxiety has been shown to have widespread effects on learning and decision-making^8–11^, its potential effect on the very first stage of learning, namely the decision to seek information, has been relatively understudied. There is evidence that when people are anxious about a specific topic or event, such as health or politics, they search for more information about that topic/event^12–16^. However, anxiety can provide a global, rather than specific, danger signal^17^. We thus hypothesized that the effects of anxiety on what people want to know may be observed even when the reason for anxiety is unrelated to the information sought (e.g., anxiety triggered by a professional conflict may impact information-seeking about finance or health).

We tested three non-mutually exclusive hypotheses of how anxiety alters information seeking. First, we tested whether anxiety is related to an increase in the general frequency of information-seeking. As knowledge can enhance a person’s sense of control, which is reduced in anxiety^18^, greater information-seeking may be a compensatory mechanism, regardless of the specific cause of the anxiety. Second, we tested whether anxiety is related to the valence of information people seek out. On one hand, anxious individuals are known to exhibit attentional biases towards negative stimuli^19–21^, suggesting that anxiety may increase the search for negative (over positive) information. On the other hand, anxious individuals need less negative information to reach negative conclusions than vice versa^22,23^. Thus, they may seek negative information less than positive information. Third, we tested whether anxiety is related to greater information-seeking in response to the magnitude of changes occurring in the environment. Anxious individuals have problems learning in changing environments^8,10^, thus they may need more information in such instances.

To test these hypotheses, we conducted three studies. First, we ran an ecological study (Study 1) during a ‘real world’ threatening event (i.e., COVID-19). Because the pandemic triggered large changes to the environment, which were also negative, it presented an ideal scenario to test whether such conditions were associated with greater information-seeking. Next, to tease apart the impact of these two variables (large changes and valence) on information seeking, we conducted two controlled laboratory experiments (Study 2). These also allowed us to examine whether and how anxiety effects information-seeking when information is unrelated to the cause of the anxiety. We were interested in effects on (1) the frequency of information-seeking, (2) the valence of information sought, and (3) whether effects were observed in response to the magnitude of changes in the environment. In one experiment (Study 2a) we examined associations between these factors and trait anxiety, while in the second experiment (Study 2b) we manipulated anxiety, thus testing for a causal link between anxiety and the above aspects of information-seeking. By dissociating the cause of the anxiety from the content of the information sought, we provide a broad perspective on the effects of anxiety on information-seeking.

## Study 1: information-seeking and anxiety in a threatening natural setting

### Methods

#### Participants

##### Time Point 1

1166 participants took part in the study by completing an online questionnaire on Prolific Academic (https://www.prolific.co/) between March 26 and 29, 2020. The sample size was determined based on our pilot study to achieve power of 0.85 of the weakest effect tested in the study (alpha = 0.05). All participants were residing in the US at the time of testing. To assure the sample was a representative sample of the US population we selected the representative samples tool available in Prolific. Prolific takes the intended sample size and stratifies it across age, sex and ethnicity according to the census data from the US Census Bureau to divide the sample into subgroups with the same proportions as the national population. The sample was representative of the US population in terms of age, sex and ethnicity (83% Caucasian, 6% African-American, 5% Asian, 4% mixed, 1% other). Participants’ engagement and attention were tested through various catch trials throughout the experiment (more details are provided in the Supplementary Material S1). 21 participants failed to select the appropriate response more than once on catch trails and therefore were excluded from the analysis. 101 participants did not indicate their ethnicity, so the final sample was composed of 1065 individuals (age = 44.78, SD = 15.49; females = 51.8%). All participants provided informed consent and were paid £3.75 for their participation. The study was approved by the UCL Research Ethics Committee and performed in accordance with relevant guidelines and regulations.

##### Time Point 2

Between April 23–25 2020, 700 of the participants recruited in Time point 1 filled in a second questionnaire on Prolific Academic (age = 46.3, SD = 15.22; females = 51.9%). No participant failed more than one catch trial (more details are provided in the Supplementary Materials S1). All participants provided informed consent and were paid £1.88 for their participation. The study was approved by the departmental ethics committee at UCL.

#### Procedure

##### Time Point 1

Participants provided an answer to this question: *“How often do you consume information on COVID-19 (news, internet *etc*..)?”* on a scale from “Never” (0) to “At least once an hour” (6) on a discrete scale (See Supplementary Materials S1 for details). State anxiety was measured with the Short State Anxiety Inventory (SSAI,^24^). We also assessed COVID-19-related anxiety by asking participants to answer on a continuous visual analogue scale ranging from 0 (none at all) to 100 (very much) the following questions: (1) *“Are you anxious about your own health?”;* (2) *“Are you anxious about the health of your loved ones in light of COVID-19?”;* (3) “*Are you anxious about dealing with lockdown in your area?”;* (4) *“Are you anxious about the consequence to your income/savings in light of COVID-19?”;* (5) *“Are you anxious about not being able to exercise?”;* (6)* “Are you anxious about not having access to food/medicine/other supplies?”*; (7) *“Are you anxious about not being able to socialize?”.*

At the same time, we collected demographic information including participants’ age, gender, current residence, level of education, household income, political orientation, ethnicity, whether they had dependents and the satisfaction with their health insurance. Additional information collected was part of other studies carried out by members of our lab (e.g.,^23,25^). We report the other questions included in the survey in the Supplementary Materials S1.

##### Time Point 2

Participants filled in the same questions as in Time Point 1. Many of the items presented in the survey completed by subjects in Time Point 1 for the parallel studies conducted in the lab were not included at Time Point 2 (see the Supplementary Materials S1 for details). Demographics were not assessed a second time.

#### Analysis

Given that the COVID-19 anxiety scores and the SSAI score were highly correlated across individual (Time Point 1: R = 0.511, p < 0.001, Time Point 2: R = 0.549, p < 0.001), we computed an aggregate ‘Anxiety Index Score’. The ‘Anxiety Index Score’ was computed by summing the COVID-19-related anxiety questions and the SSAI score (the SSAI score was considered as one measure) and dividing by the total number of questions. The SSAI scores were transformed to the same range as the COVID-19-related anxiety questions (0–100). Then, we ran a linear regression predicting COVID-related information-seeking ratings from the Anxiety Index Score, adding all demographics as covariates (age, gender, educational level, income, political orientation, ethnicity, whether they had dependents and the satisfaction with their health insurance). Importantly, however, we also ran the same analysis with either the average of the COVID-19-related anxiety questions or the SSAI score separately as independent measures, with all demographics as covariates. All the analyses were repeated separately for time 1 and 2. Group-level linear regressions were carried out with R (4.0.1) using the lm package.

### Results: anxiety was associated with increased information-seeking about COVID-19

**Figure 1:**
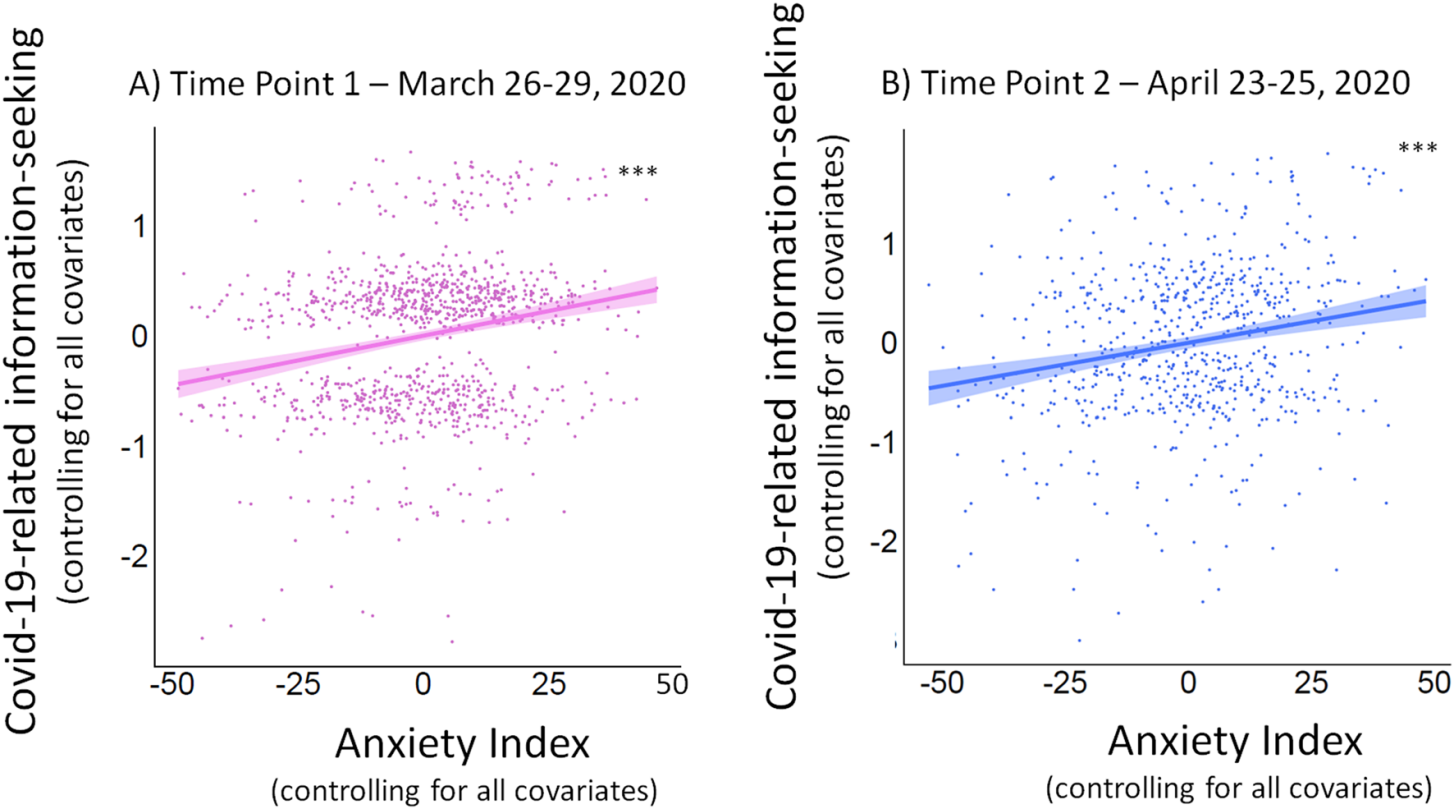
Individuals reporting greater Covid-19-related anxiety sought more Covid-19-related information (Study 1). Plotted are the partial linear regressions predicting self-reported Covid-19-related information-seeking (obtained by asking participants *“How often do you consume information on COVID-19 (news, internet *etc*..)?”* on a scale from “Never” (0) to “At least once an hour” (6) on a discrete scale) from the Anxiety Index Score (obtained by summing the COVID-19-related anxiety questions and the SSAI score and dividing by the total number of questions) at Time Point 1 (**A**; p < 0.001) and Time Point 2 (**B**; p < 0.001), controlling for age, gender, level of education, household income, political orientation, ethnicity, whether they had dependents and the satisfaction with their health insurance. Both graphs show that individuals with higher Anxiety Index Score report engaging more in COVID-related information seeking. ***p < 0.001. (Note that the observed clustering along the Y-axis in both graphs is due to the discrete nature of the rating scale used to measure Covid-19-related information-seeking).

Our findings suggest that anxiety was related to increased information-seeking during a ‘real world’ threatening event. Specifically, participants with higher Anxiety Index Score reported engaging more in information-seeking about the COVID-19 pandemic at Time Point 1 (β = 0.009 ± 0.001, p < 0.001, $\eta_p^2$ = 0.045; Fig. 1A) and Time Point 2 (β = 0.008 ± 0.002, p < 0.001, $\eta_p^2$ = 0.039; Fig. 1B). The same results were also obtained when the average of the COVID-19-related anxiety questions was included as the only predictor of information-seeking (Time Point 1: β = 0.008 ± 0.001, p < 0.001, $\eta_p^2$ = 0.037; Time Point 2: β = 0.008 ± 0.002, p < 0.001, $\eta_p^2$ = 0.035;) or when the SSAI score was included as the only predictor (Time Point 1: β = 0.011 ± 0.001, p < 0.001, $\eta_p^2$ = 0.05; Time Point 2: β = 0.009 ± 0.002, p < 0.001, $\eta_p^2$ = 0.028).

Results from Study 1 suggest that individuals who reported higher anxiety were more likely to seek information related to the pandemic. Different features of the environment may have influenced information-seeking. These features included large change in the environment and negative valence. In order to tease apart these features, we conducted two controlled laboratory experiments (Study 2). By manipulating the magnitude and direction of changes in the environment, we examined how anxiety effected the valence of information sought, and whether effects were observed selectively in response to large changes in the environment.

## Study 2a: trait anxiety and information-seeking

### Methods

#### Participants

We based our sample size on a previous study from our lab in which anxiety was manipulated exactly as done in the current study^22^. In that paper, a linear regression was conducted predicting behavior from anxiety change similarly to what we did in the current study. The effect size was $\eta_p^2$ = 0.25 (equivalent to f^2^ = $\eta_p^2$/(1 − $\eta_p^2$) = 0.33). Using G*Power we estimated the required sample size (alpha = 0.05, beta = 0.85, 3 original predictors) as 42, and we added a 10% drop out rate to that number. This sample size is also consistent with other previous studies investigating the role of trait anxiety^8,26–28^ or induced anxiety^10,29^ on learning and decision-making. The sample size in those studies range from 20 to 50.

45 participants were recruited. Data from participants who missed more than 25% of all trials were excluded (N_excluded_ = 3). Final sample was then of 42 participants (15 males, 27 females, mean age = 28.02 years ± 11.62 (SD), age range: 18 to 66 years). All participants were recruited via the UCL subject pool, and the experiment was approved by the departmental ethics committee at UCL.

#### Procedure

##### Behavioral task

**Figure 2:**
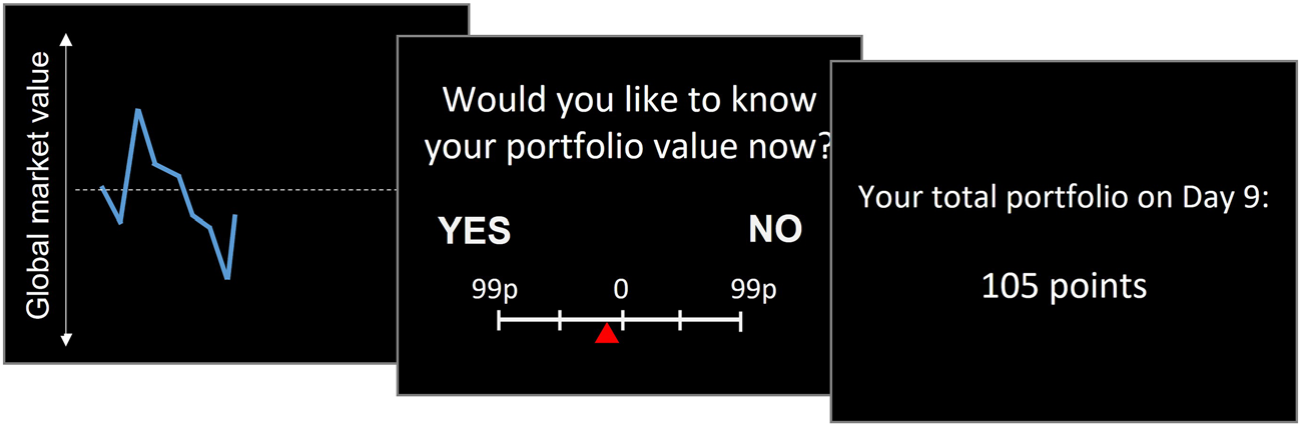
Stock market task. Participants observed the market going up or down on each trial. They were then asked whether they want to find out the value of their portfolio and had 8 s to indicate this using a willingness to pay scale. Specifically, the more they paid towards ‘YES’ the more likely they would be to receive information, and the more they paid towards ‘NO’ the more likely they would remain ignorant. They then received feedback for 3 s with either the value of their portfolio or ‘XX points’.

Participants played a Stock Market Task^30,31^ (Fig. 2). This task consisted of 4 blocks of 50 trials each. At the beginning of each block, participants were endowed with 100 points, worth ₤10, which they were to invest in 2 of 5 fictitious stocks that compose a ‘global market’. On each trial participants observed the global market evolution (a dynamic increase or decrease in the curve lasting 2.3 s). Altering the direction and magnitude by which the market was changing from trial to trial allowed us to manipulate both the expected valence of the information (likely positive gain or negative loss) and the degree of change. Given that the participant’s portfolio consisted of 2 companies out of the 5, the global market was a partial indicator of the change to the participant’s own portfolio value. Unbeknown to the participants, on each trial there was a 65% likelihood that their portfolio would follow the market trend; otherwise, the portfolio would vary in the opposite direction than the market with a randomly-generated magnitude.

On each trial, after observing the change to the global market, participants bid for a chance to know (or remain ignorant about) the value of their portfolio (Fig. 2). In particular, they had 8 s to indicate how much they were willing to pay to receive this information or to avoid it. They did so using a scale ranging from 99p to avoid information, through 0p (no preference), to 99p to receive information (left/right positions of ‘YES’ information and ‘NO’ information were counterbalanced across participants). We refer to this scale as the Willingness To Pay (WTP) scale^32^. Participants were told that the more they were willing to pay, the greater the probability that their decision (to receive or avoid information) would be honored. If they selected ‘0p’, then information was delivered at random (50%). If they selected an amount between 1 and 20p, their wish was honored with 55% probability; between 21 and 40p, their wish was honored with 65% probability; and so on up to 95% probability. Participants were not aware of how exactly each WTP response was converted in the probability of receiving information. Next, their portfolio value in points was presented on screen for 3 s or hidden (‘XX points’ was shown). Information was non-instrumental; it could not be used to increase rewards, avoid losses, or make alterations to portfolio. In the last two blocks (blocks 3–4**),** after they observed the global market, participants were asked to answer two additional questions which are beyond the scope of this study (more details in the Supplementary Materials S1).

##### Incentive-compatible payment

At the end of the task one trial was selected at random (regardless of whether information was delivered) for payment. Participants would receive their portfolio value on that trial. If they paid money to receive or avoid information on that trial and their choice was respected, that amount was deducted from their payment (e.g. if they earned 110 points and selected to pay ₤0.50 to receive information which they did, then they would get ₤11 − ₤0.50 = ₤10.50).

##### Trait anxiety

Finally, participants completed the State-Trait Anxiety Inventory (STAI). Mean trait anxiety was 42.21 ± 11.69 (SD) and ranged from 20 to 68. Some of the task-related data was previously published in Ref.^30^—the relationship between information-seeking tendencies and trait anxiety however had never been assessed before in that dataset nor previously published.

#### Analysis

**Figure 3:**
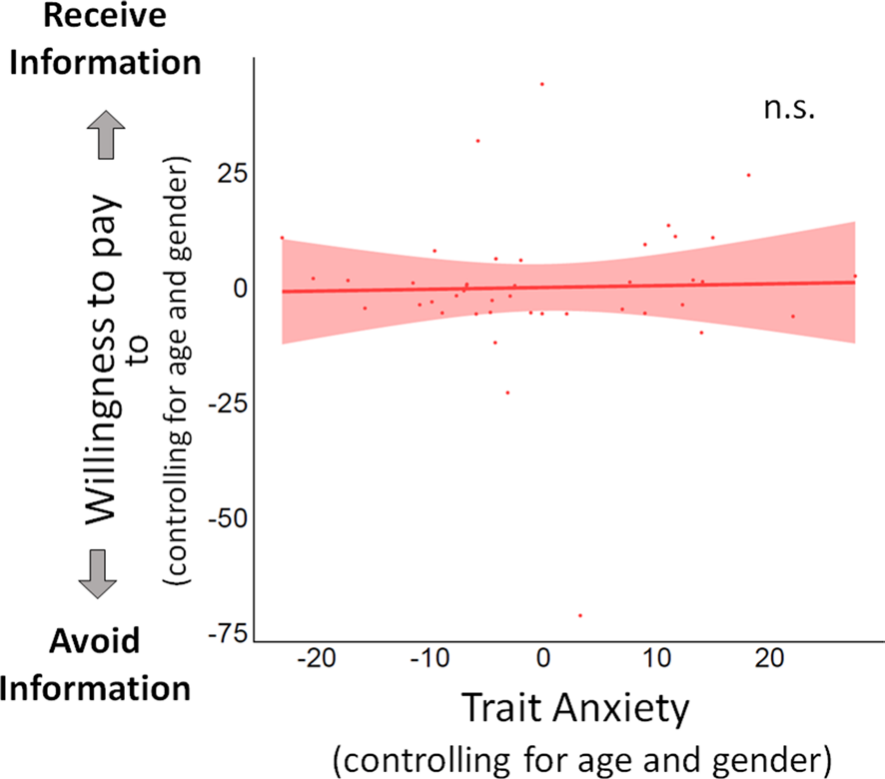
Trait anxiety was not related to willingness to pay for information (Study 2a). Plotted is the partial linear regression predicting WTP (in pence, coded positively if participants indicated they wanted to receive information and negatively if they wanted to avoid information) from trait anxiety (State-Trait Anxiety Inventory), controlling for age and gender (p = 0.863). Results did not change when removing the one outlier. Also note that plotted values are residuals after controlling for age and gender, hence the negative trait anxiety values.

**Figure 4:**
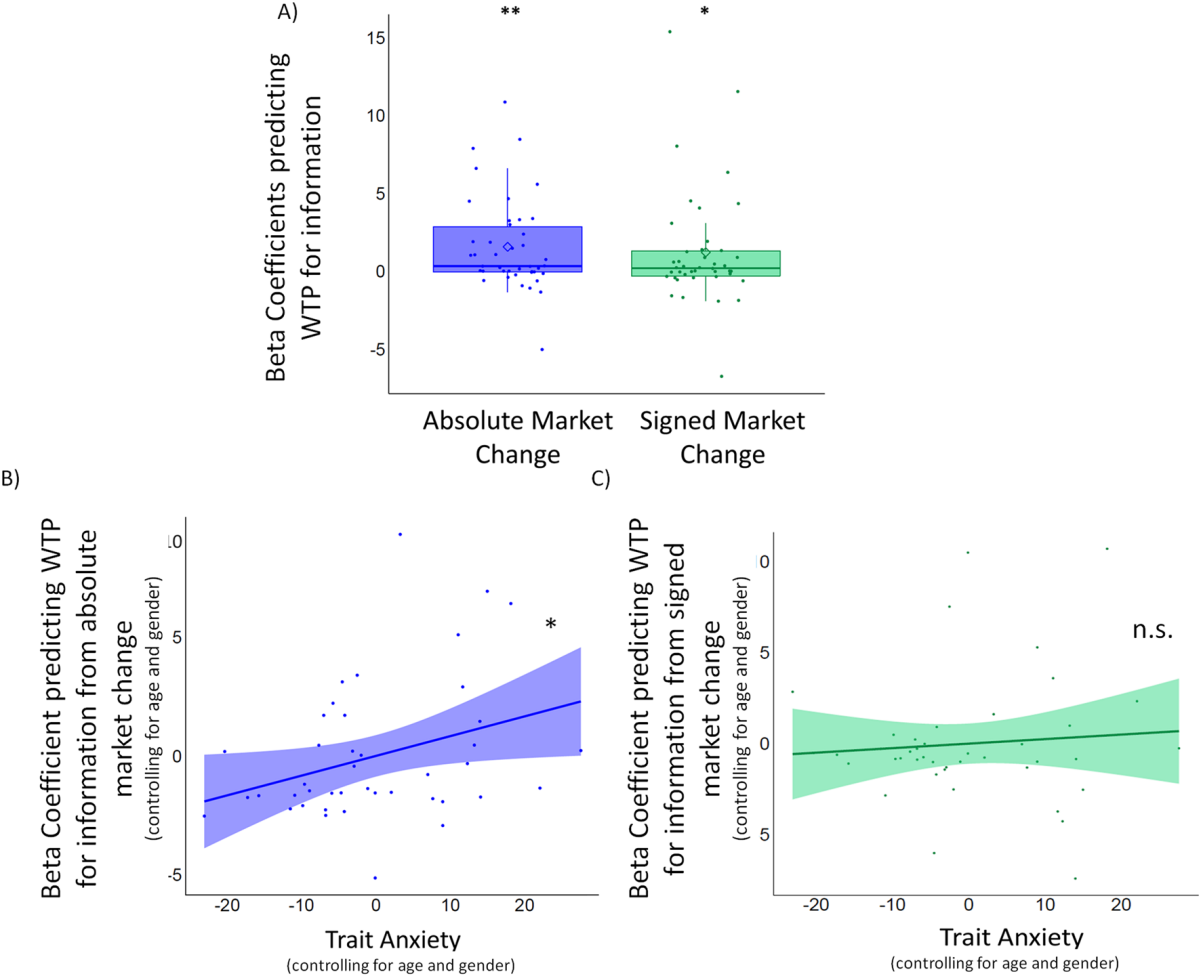
Trait anxiety amplifies the impact of the magnitude of change in the environment on information-seeking (Study 2a). (**A**) A linear regression was conducted for each participant to predict WTP from the signed market change and absolute market change and the resulting betas were then compared to zero with a one-sample t-test. Both signed market change (signaling whether the market was going up or down) and absolute market change (signaling the magnitude of changes in the market) predicted participants’ willingness to pay for information. Specifically, participants’ willingness to pay for information was greater when the market was going up rather than down and when there were large changes in the market. Plotted are the beta coefficients reflecting the effect of signed (green) and absolute (purple) market change on WTP. Horizontal lines indicate median values, boxes indicate 25–75% interquartile range, diamonds indicate mean values and whiskers indicate 1.5 × interquartile range; individual coefficients are shown separately as dots. **p = 0.01, *p < 0.05. (**B**,**C**) Increased trait anxiety was associated with greater impact of absolute market change on information-seeking (p = 0.039) (**B**) but not with changes to the impact of valence on information-seeking (p = 0.616) (**C**). (**B**,**C**) Plotted are the partial linear regressions predicting the beta coefficients shown in (**A**) from trait anxiety, controlling for age and gender. Also note that in panels (**B**) and (**C**) plotted values are residuals after controlling for age and gender, hence the negative trait anxiety values.

First, we computed willingness to pay (WTP) on every trial, with amount paid to avoid information scored negatively, and amount paid to receive information positively (zero is simply coded as zero). We then investigated how trait anxiety was related to information-seeking behavior. We first performed a group-level linear regression to predict participants’ average WTP from trait anxiety scores, controlling for age and gender (Fig. 3, Table S7). We then focused on whether specific factors that drive information-seeking were modulated by trait anxiety. To do so, we performed individual-level linear regressions to predict, for each participant, WTP for information on each trial from two factors (z-scored) we have previously shown to impact information-seeking in this task^30,31^: (1) valence (quantified as signed market change, which is the amount by which the market went up or down) and (2) absolute market change, which represents the magnitude of changes in the environment, independent of valence. The resulting betas were then compared to zero with a two-tailed one-sample t-test (Fig. 4A). We then performed two separate group-level linear regressions predicting each beta coefficient from trait anxiety, controlling for age and gender by adding them as covariates in the regression (Fig. 4B,C, Tables S8, S9). Group-level linear regressions were carried out with R (4.0.1) using the lm package, while individual-level linear regressions were carried out with Matlab (R 2019a) using the function glmfit. All statistical tests were two-tailed.

We confirmed the results of the above analyses using a Linear Mixed Effects model predicting willingness to pay for information. The model included the following fixed effects: absolute market change, signed market change, trait anxiety, age, gender and the interactions between absolute market change and trait anxiety and signed market change and trait anxiety (Table S13). Absolute and signed market change were also modelled as random effects. Intercept was included as both fixed and random effects. Variables were z-scored before running the Linear Mixed Effects models. All linear mixed models were run in R using the lmer function (lme4 package) using maximum likelihood estimation method, the BOBYQA (Bound Optimization BY Quadratic Approximation) optimizer and a maximum number of iterations of 100,000.

### Results

#### Trait anxiety was not associated with increased frequency of information-seeking

We first tested whether trait anxiety was related to a general increase in information-seeking by examining whether the Willingness To Pay (WTP) for information was related to anxiety levels. WTP was coded positively if participants indicated they wanted to receive information and negatively if they wanted to avoid information. We ran a linear regression to predict participants’ average WTP from trait anxiety scores controlling for age and gender (Table S7). Results showed that anxiety levels did not predict WTP (β = 0.039 ± 0.23, p = 0.863, $\eta_p^2$ = 0.001; = Fig. 3). This suggests that trait anxiety was not related to general changes in information-seeking.

#### Trait anxiety was associated with greater information-seeking in response to large magnitude (but not the valence) of changes

A linear regression was conducted for each participant to predict WTP from the signed market change and absolute market change (z-scored) and the resulting betas were then compared to zero with a two-tailed one-sample t-test. Consistent with previous studies^30,31^, we found that participants’ WTP for information was greater when the market was going up rather than down (*signed market change:* β = 1.23 ± 0.56, t(41) = 2.17, p = 0.035) and when the magnitude of changes in the market increased (*absolute market change:* β = 1.58 ± 0.46, t(41) = 3.46, p = 0.001; Fig. 4A).

We then performed two linear regressions to predict each of the two beta coefficients (signed and absolute market change) from trait anxiety scores, controlling for age and gender. Results revealed that greater trait anxiety was related to an increase in the WTP for information when there were larger changes in the market (β = 0.08 ± 0.04, p = 0.039, $\eta_p^2$ = 0.108; Fig. 4B, Table S8), and was unrelated to the impact of valence on information-seeking (β = 0.025 ± 0.05, p = 0.616, $\eta_p^2$ = 0.007; Fig. 4C, Table S9).

Overall, results from Study 2a suggest that individual differences in trait anxiety were related to differences in the desire for information as a function of magnitude of changes in the environment. Anxiety was not associated with the frequency of information-seeking per se, nor with valence-dependent information-seeking. Higher trait anxiety appeared to be selectively associated with increased information-seeking in response to larger changes in the environment.

To confirm the above results we conducted a Linear Mixed Effects model, which predicted willingness to pay for information from the following fixed effects: absolute market change, signed market change, trait anxiety, age, gender, the interactions between absolute market change and trait anxiety and signed market change and trait anxiety. Absolute and signed market change were also modelled as random effects. Intercept was included as both fixed and random effects. (see Methods for details). Results showed significant main effects of signed (β = 1.24, SE = 0.55, t(42.26) = 2.25, p = 0.029) and absolute market change (β = 1.60, SE = 0.43, t(41.64) = 3.68, p < 0.001). The interaction between trait anxiety and absolute market change was also significant (β = 0.93, SE = 0.43, t(41.06) = 2.15, p = 0.036). No other effects were significant (see Table S13 for full results). This replicates our previous findings using a two-step procedure where the impact of absolute market change on willingness to pay was influenced by trait anxiety scores. The results suggest that anxiety magnifies the impact of market changes on information-seeking, but has no effect on overall information-seeking (no main effect of anxiety) or on the impact of valence (no anxiety by signed market change interaction).

## Study 2b: induced anxiety and information-seeking

Study 2a revealed that trait anxiety amplified the impact of changes to the market on information-seeking. We next tested for the causality of this relationship by manipulating individuals’ anxiety levels and measuring their information-seeking behavior.

### Methods

#### Participants

Fifty participants were recruited via the UCL subject pool and the experiment was approved by the departmental ethics committee at UCL. One participant did not complete the experiment and data from another participant was lost. Final data are thus reported on 48 participants. The induced anxiety group was composed of 24 participants (10 males, 14 females, mean age = 21.96 years ± 3.51 (SD), age range: 19 to 32 years) and the control group of 24 participants (10 males, 14 females, mean age = 23.46 years ± 4.79 (SD), age range: 19 to 34 years). There was no age difference between groups (t(46) = 1.238, p = 0.22). Sample size was calculated as detailed in Study 2a.

#### Procedure

##### Behavioral task

Participants performed the same task as in Study 2a (Fig. 2).

##### Induced anxiety

Before the task, participants were randomly assigned to one of two groups: *induced anxiety* group and *control* group. The *induced anxiety* group was administered a modified version of the Trier Social Stress Test (TSST)^22,33–35^. Specifically, they were told that at the end of the task, they would have to give a 5-min presentation on a surprise topic in front of a panel of senior academics and that they would be videotaped and judged during that presentation. The main difference with the typical TSST is that participants were threatened by a stressful social event and completed the task under that threat, but the threat was in fact never executed. In addition, they were given six difficult math problems to solve in less than 30 s before the beginning of the task. Participants assigned to the *control* group were told they would have to write a short essay at the end of the task, but would not be judged. They were also given six very easy math problems to solve in 30 s. To measure whether the manipulation was effective, all participants completed the Short State Anxiety Inventory (SSAI)^24^ before and after the manipulations described above. We then used these questionnaires to compute the change in anxiety levels by subtracting the anxiety score reported before the stress or control manipulation from the anxiety score reported after the manipulation. Using change in SSAI as a measure of task-induced anxiety allowed individual variations in response to the TSST to be taken into consideration, as it is known that responses to the TSST vary widely among individuals depending on age^36^, gender^37^, education^38^, personality traits^39^, use of nicotine, alcohol and caffeine^36^ and use of medications^40^ (for an overview of individual differences in TSST performance, see^36^).

#### Analysis

Analysis was performed as in Study 2a with the following differences:

1. Short *State* Anxiety Inventory scores were used to measure anxiety.
2. Group (control/induced anxiety) was added to both the Linear Regressions and the Linear Mixed Models.
3. The Linear Mixed Model was identical to the one run for Study 2a, but using change in anxiety instead of trait anxiety and group was added as fixed effects (whether participants were in the control group or the induced anxiety group) (Table S14).

### Results

#### Anxiety levels were successfully manipulated

**Figure 5:**
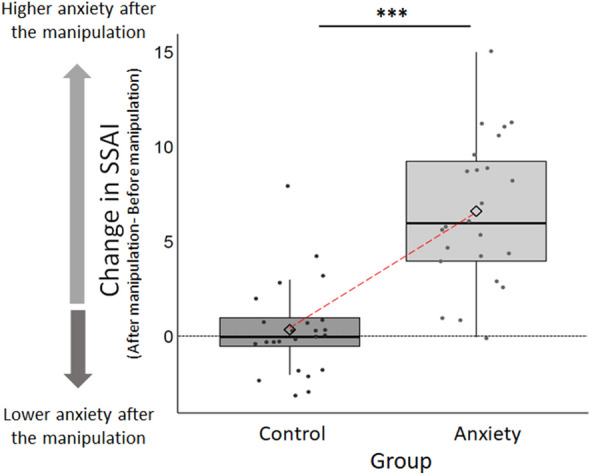
Change in anxiety levels was stronger in the *induced anxiety* group as compared to the *control* group (Study 2b). Plotted is the change in SSAI induced by the manipulation in both groups. Change in anxiety was computed for each individual by subtracting the SSAI score before the manipulation from the SSAI score after the manipulation. Horizontal lines indicate median values, boxes indicate 25–75% interquartile range, diamonds indicate mean values, the horizontal dotted line indicate 0 change in anxiety scores and whiskers indicate 1.5 × interquartile range; individual scores are shown separately as dots. ***p < 0.001.

The anxiety manipulation was successful. After the manipulation, individuals in the *induced anxiety* group exhibited greater anxiety, as measured by the SSAI scores (N = 24, mean SSAI score = 16.42, SD = 4.35), than individuals in the *control* group (N = 24, mean SSAI score = 10.46, SD = 4.00) (difference: t(46) = 4.93, p < 0.001), with no difference between groups before the manipulation (*induced anxiety* group mean SSAI score = 9.79, SD = 3.93; *control* group mean SSAI score = 10.08, SD = 3.78; group difference: t(46) = 0.26, p = 0.794). The change in reported anxiety (after minus before the manipulation) was significantly higher in the *induced anxiety* group compared to the *control* group (*induced anxiety* group mean SSAI score change = 6.63, SD = 3.83, *control* group mean SSAI score change = 0.38, SD = 2.43; group difference: t(46) = 6.75, p < 0.001, Fig. 5).

There was substantial variability in individual susceptibility to the manipulation, with some individuals reporting increased anxiety after the control manipulation, and others individuals reporting very little change in anxiety after the anxiety manipulation^36,37,39^. To account for individual differences in response to the manipulation, and for consistency with Study 2a, we therefore used the change in SSAI as a predictor of information-seeking behavior in all analyses.

#### Induced anxiety did not alter overall willingness to pay for information

**Figure 6:**
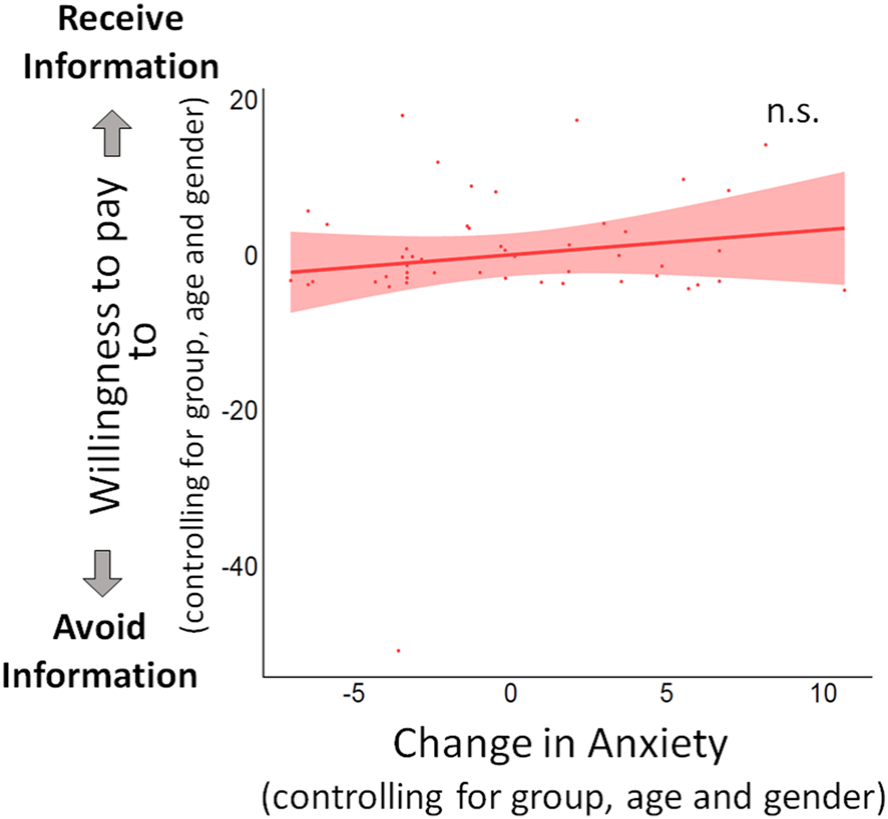
Change in anxiety was not related to willingness to pay for information (Study 2b). Plotted is the partial linear regressions predicting WTP (in pence, coded positively if participants indicated they wanted to receive information and negatively if they wanted to avoid information) from change in anxiety induced by the manipulation, controlling for age and gender (p = 0.437). Results did not change when removing the one outlier.

The results showed that WTP for information was unrelated to induced anxiety. In particular, a linear regression predicting WTP from change in SSAI, controlling for group, age and gender (Table S10), was not significant (β = 0.358 ± 0.46, p = 0.437, $\eta_p^2$ = 0.014; Fig. 6). Consistent with results from Study 2a, this result suggested that anxiety did not induce a general change in information-seeking.

#### Induced anxiety led to greater information-seeking in response to the large magnitude (but not valence) of changes

**Figure 7:**
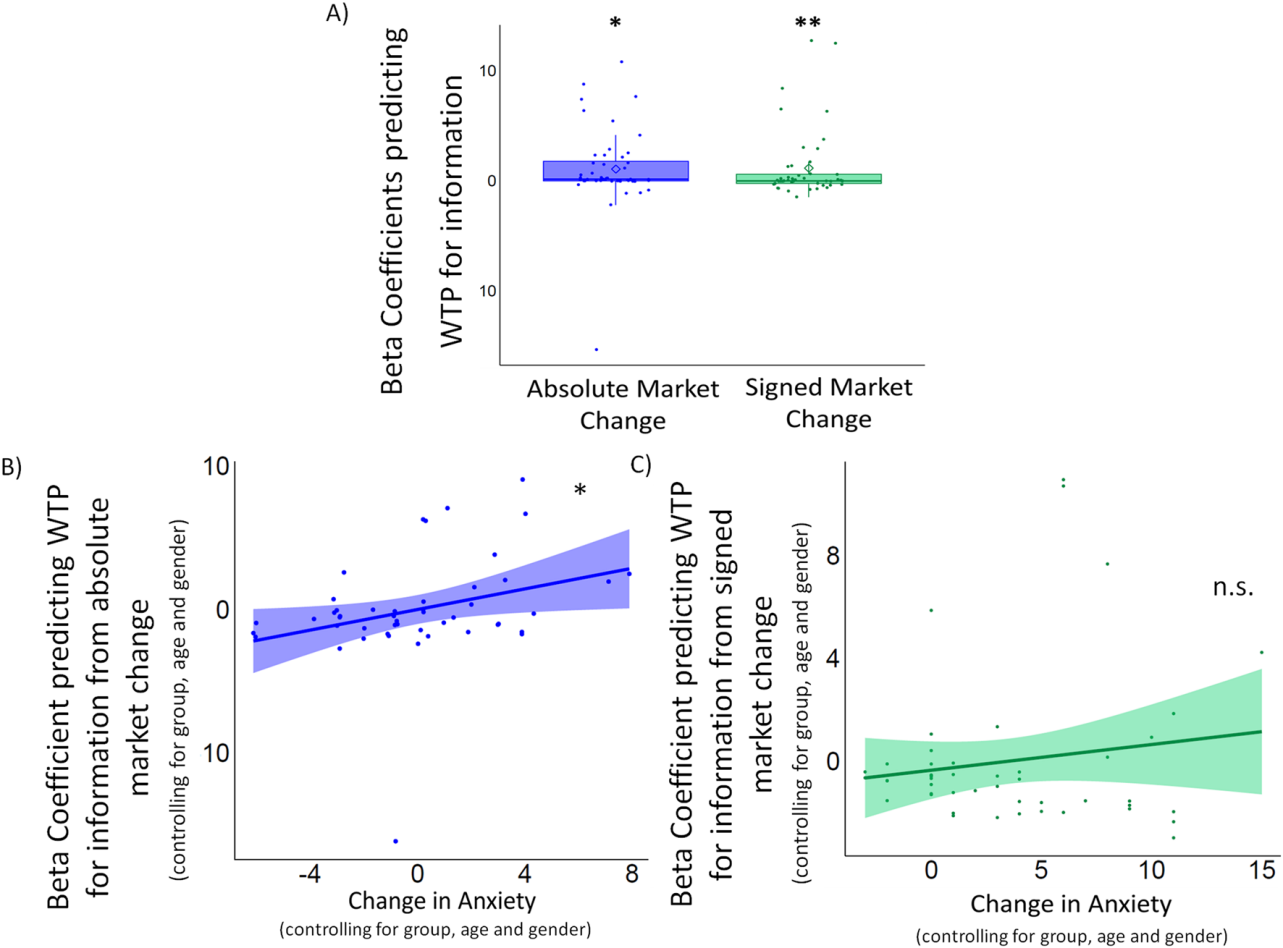
Induced anxiety led to greater information-seeking when the magnitude of changes in the environment increased, regardless of valence (Study 2b). (**A**) Both signed market change (signaling whether the market was going up or down) and absolute market change (signaling the magnitude of changes in the market) predicted participants’ willingness to pay for information. Plotted are the beta coefficients reflecting the effect of signed (green) and absolute (purple) market change on WTP. Horizontal lines indicate median values, boxes indicate 25–75% interquartile range, the crosses indicate mean values and whiskers indicate 1.5 × interquartile range; individual scores are shown separately as circles. ** p = 0.01, * p < 0.05. (**B**) Induced anxiety resulted in greater influence of magnitude of market change (p = 0.037; results do not change when excluding the one outlier). (**C**) Induced anxiety was not associated with the effect of valence of market change on information-seeking (p = 0.16). (**B**,**C**) Plotted are the partial linear regressions predicting the beta coefficients shown in (**A**) from change in anxiety, controlling for group, age and gender.

As in Study 2a, we examined whether anxiety altered the impact of signed and absolute market change on information-seeking. First, a linear regression was conducted for each participant to predict WTP from the signed market change and absolute market change and the resulting betas were than compared to zero with a two-tailed one-sample t-test. Again, we found that participants’ willingness to pay for information was greater when the market was going up rather than down (*signed market change:* β = 1.15, t(47) = 2.57, p = 0.013) and when there were larger changes in the market (*absolute market change:* β = 1.06, t(47) = 2.02, p = 0.049, same is true when excluding the one outlier: β = 1.41, t(46) = 3.51, p = 0.001) (Fig. 7A).

Next, we investigated whether the influence of these two features on information-seeking was altered by induced anxiety. To that end, we ran two linear regressions to predict the beta coefficients obtained in the previous analysis for signed and absolute market change from changes in SSAI, controlling for group (induced anxiety or control group), age and gender. Similar to what we found for trait anxiety in Study 2a, results showed that induced anxiety increased the impact of absolute market change on information-seeking (β = 0.359 ± 0.167, p = 0.037, $\eta_p^2$ = 0.097; Fig. 7B, Table S11; the same results were obtained when excluding the one outlier: β = 0.329 ± 0.12, p = 0.009, $\eta_p^2$ = 0.150).

This result conceptually replicated the finding from Study 2a (with induced anxiety instead of trait anxiety), and provided evidence that anxiety *causally* increased information-seeking about large changes in the environment. In addition, similarly to Study 2a, we found that anxiety was not associated with valence-dependent information-seeking (β = 0.205 ± 0.144, p = 0.16, $\eta_p^2$ = 0.045; Fig. 7C, Table S12).

As in Study 2a, we then confirmed these results adopting a multilevel approach (see Methods for details). Results showed significant main effects of signed (β = 1.13, SE = 0.41, t (48.86) = 2.74, p = 0.008) and absolute market change (β = 1.03, SE = 0.49, t (47.52) = 2.10, p = 0.04). The interaction between change in anxiety and absolute market change was also significant (β = 1.09, SE = 0.49, t (47.56) = 2.22, p = 0.03). Here we also found a significant interaction between change in anxiety and signed market change (β = 0.94, SE = 0.41, t (49.09) = 2.28, p = 0.02). This result is neither consistent with what we obtained with the previously adopted analysis method nor with the results obtained in Study2a, therefore it should be interpreted with caution. No other effects were significant (see Table S14 for full results). These results again suggest that the impact of absolute market change on information-seeking was influenced by induced anxiety. Specifically, anxiety did not directly increase information-seeking, but rather magnified the impact of market changes on information-seeking.

In sum, our results suggest that anxiety did not alter the overall tendency to seek or avoid information, nor did it impact valence-dependent information-seeking, but rather it specifically magnified the impact of the magnitude of changes in the environment on information-seeking both in Study 2a (trait anxiety) and Study 2b (induced anxiety).

## Discussion

In the present study we shed light on the relationship between anxiety and information-seeking. We show that anxiety led to increased information-seeking in response to larger changes, rather than to a general increase in the desire for information. This was true even when the cause of the anxiety (e.g., a stressful social situation) was not related to those changes (e.g., changes are financial). This suggests that the influence of anxiety on information-seeking can “spill over” to other aspects of one’s life that are not necessarily related to the source of anxiety.

In addition, results from our ecological study show that participants who reported greater anxiety during the pandemic sought more information about COVID-19, consistent with past studies (e.g.,^14,41,42^). The pandemic triggered large changes in the environment and negative valence, which may underlie the increase in information-seeking.

We were able to fully dissociate these drives in controlled laboratory experiments. In particular, results from our information-seeking task suggest that participants with higher trait anxiety exhibited a selective increase in the impact of large change in the environment on information-seeking. These results were replicated in a second study where anxiety was experimentally induced, providing causal evidence for this relationship. We did not find evidence that anxiety was related to the impact of valence on information-seeking in either study. These laboratory results could speak to the findings of our ecological study, suggesting that large changes in the environment during the pandemic, rather than the negative valence of the news, may have contributed to increased information-seeking in anxious individuals.

Heightened information-seeking in response to large changes may be adaptive. When experiencing a major life change (e.g. moving countries, changing jobs, etc.), seeking information helps adapt to the new environment. Interestingly, however, in our lab studies, this effect was present even though the cause of the anxiety was unrelated to the information sought. For example, in Study 2b, inducing anxiety by exposing participants to a stressful social situation, led to greater desire for information about the value of stocks when the financial market was experiencing larger changes. This finding may reflect a compensatory mechanism. In particular, anxious individuals have difficulty learning when in volatile environments^8,10^, are more averse to ambiguity^43^ and generally misestimate the levels of risk and uncertainty when learning and making decisions^44,45^. They may thus require more information to adapt their behavior in changing environment and reduce uncertainty. This tendency, however, may become maladaptive if the information is predominantly negative, leading to low mood^4,5,7,46^, as well as increased stress and psychopathology symptoms^6,47,48^.

Previous studies investigating information-seeking and psychopathology suggest that different clinical populations may be characterized by different information-seeking patterns. For example, it has been suggested that individuals who suffer from social anxiety disorders look for less information before making social ranking decisions^49^ while high obsessive–compulsive individuals seek more information in general^42^ and need more evidence to make decisions^9^. It would be useful to extend the study of information-seeking in highly changing environments to include clinically anxious participants to examine whether the effects reported here replicate in a clinical population.

Taken together, our findings shed new light on the intricate relationship between anxiety and information-seeking by showing a link between anxiety and increased information-seeking in response to large magnitude of change. When facing large changes in their environment, such as switching jobs or moving cities, anxiety may drive individuals to search for more information. On one hand, this may reduce uncertainty and increase adaptation, but in extreme cases may lead to indecision and information overload.

## Supplementary Information

Supplementary Information.

## Data Availability

Anonymized data and code is available at this link: https://github.com/affective-brain-lab/Anxiety-increases-information-seeking-in-response-to-large-changes-.git.
