# Supplementary material for: Anxiety increases information-seeking in response to large changes
Source: Sci Rep. 2022 May 5;12:7385. doi: 10.1038/s41598-022-10813-9 (PMC9070976; doi:10.1038/s41598-022-10813-9)
Supplement: Supplementary file 1 — Supplementary Information. [file 41598_2022_10813_MOESM1_ESM.docx]

**Anxiety increases information-seeking in response to large changes**

Caroline Charpentier*^1-2^, Irene Cogliati Dezza*^3-5^, Valentina Vellani*^3-4^, Laura K. Globig^3-4^, Maria Gädeke^6^, Tali Sharot^3-4^

**Affiliations:**

^1^ Division of Humanities and Social Sciences, California Institute of Technology, Pasadena, CA, USA.

^2^ Institute of Cognitive Neuroscience, University College London, London, UK.

^3^ Department of Experimental Psychology, University College London, London, WC1H 0AP, UK.

^4^ The Max Planck UCL Centre for Computational Psychiatry and Ageing Research, University College London, London, WC1B 5EH, UK

^5^ Department of Experimental Psychology, Ghent University, B-9000 Ghent, BE

^6^ Division of Medical Psychology, University of Bonn, 53113 Bonn, Germany

*These authors contributed equally.

Corresponding authors: Caroline Charpentier ccharpen@caltech.edu, Tali Sharot t.sharot@ucl.ac.uk

**Supplementary Materials**

**Supplementary Methods**

**Information collected in the questionnaire at Time Point 1**

**COVID-19-related Information-seeking:** Participants were asked to indicate how often they consumed information on COVID-19 (news, internet etc…) on the following scale: 1 (Never), 2 (Less than once a week), 3 (At least once a week), 4 (Once a day), 5 (At least 4 times per day), 6 (At least once an hour).

**Demographics**: Participants were asked to indicate their age, gender, ethnicity, current place of residence, level of education, household income, health insurance satisfaction, political orientation and whether they had children.

**Covid-19 related anxiety:** Participants who declared to have children also answered to the following question “Are you anxious about homeschooling in light of COVID-19?” on a continuous visual analogue scale ranging from 0 (none at all) to 100 (very much). This question has not been included in the main analysis as only the 22.36% of subjects indicated to have children.

**Additional information collected in the questionnaire at Time Point 1**

These additional measures are part of parallel studies conducted in our lab (Globig et al., 2020; Blain et al., 2021):

**Impact of COVID-19 on income:** Participants were asked to indicate what was the impact of COVID-19 on their income on a scale from 1 (None) to 6 (Extremely high).

**Happiness:** In order to assess happiness, participants were asked the following questions: (i)“Taken all together, how happy are you with your life these days? Mark your rating relative to the least and most happy time of your life.” Participants were asked to respond on a continuous visual analogue scale ranging from 0 (least happy time of your life) to 100 (most happy time of your life). (ii) “Think about right now. How happy are you at this moment?”. Participants were asked to respond on a continuous visual analogue scale ranging from 0 (very unhappy) to 100 (very happy).

**Emotions:** Participants were asked to report whether in the last 24 hours they felt fear, hopeful, joyful, sad, angry and surprised on a continuous scale ranging from “Not at all” to “Very much”.

**Optimism about getting COVID-19:** Participants were asked to indicate: “Relative to others of your age and gender do you think you are less/more likely to get COVID-19?” on a scale from 1 (much less likely) to 5 (much more likely).

**Perceived danger to humanity:** Participants were asked to indicate: “Do you think COVID-19 presents a real danger to the health of the human population?” on a continuous visual analogue scale from 0 (not really) to 100 (extreme danger).

**Compliance measures:** Participants rated on a continuous visual analogue scale ranging from 0 (none at all) to 100 (a lot): a) “How much effort do you make to wash your hands regularly?”; b) “How much effort do you make to socially distance yourself from others?”; c) “How much effort do you make to avoid touching your face?”, as well on a continuous visual analogue scale ranging from 0 (zero) to 100 (many times) d) “In the past week how many times have you been to another person's house ?”; e) How many days this week have you been closer than 1 meter to another person (except those you live with)?”.

**Sense of agency:** Participants completed a questionnaire assessing sense of agency which was composed by 2 parts assessing personal mastery and perceived constraints. (Lachman & Weaver, 1998).

**Behavioral Changes:** To assess behavioral changes caused by the pandemic participants were asked to indicate the frequency of face-to-face interaction, online/telephone interaction, physical activity, outdoor activity, visiting places of religious worship, social connectedness before and after the restrictions. Behaviors were reported on the same scales for before and after the restrictions.

**Addictive behaviors:** Participants reported frequency of habitudinal and addictive behaviors including smoking, alcohol consumption, gambling, eating before and after the restrictions.

**Psychopathology:** Participants completed the: Obsessive-Compulsive Inventory – Revised (OCI-R, Foa, Kozak, Salkovskis, Coles, & Amir, 1998), Patient Health Questionnaire (PHQ-9, Kroenke, Spitzer, & Williams, 2001), Apathy Evaluation Scale (AES, Mann, 1990), Life Orientation Optimism Test (LOT-R, Molina et al., 2013).

**Psychosocial Questionnaires :** Participants also completed a series of psychosocial questionnaires assessing empathic concern (Davis, 1983), resilience (Smith et al., 2008), narcissism (Leckelt et al., 2018), risk-taking propensity (GRiPS, Zhang, Highhouse, & Nye, 2019).

**Stress Coping:** Participants responded on a 5-point Likert Scale from 1 (strongly disagree) to 5 (strongly agree) to the following items: 1) The effects of stress are negative and should be avoided. 2) The effects of stress are positive and should be utilized.

**Health Anxiety:** Participants were asked to indicate their health anxiety on a 5-point Likert Scale from 1 (very inaccurate) to 5 (very accurate): Often I am concerned about diseases I might have.

**Non-Conformity:** Participants were asked to indicate conformity on a 5-point Likert Scale from 1 (very strong disagreement) to 5 (very strong agreement): I prefer to make my own way in life rather than find and follow.

**Social Support and Connectedness:** Participants were asked to indicate on a 7-point Likert Scale from 1 (strongly disagree) to 7 (strongly agree): My friends/family give me the support I need. We presented participants with a modified version of the “inclusion of others in the self” scale (Aron, Aron, Tudor, & Nelson, 1991).

**Behavioral Economic Tasks:** Participants completed a series of established behavioral tasks including a one-shot dictator game (Kahneman, 1986), an intertemporal choice task (Kirby & Maraković, 1996) and a loss aversion task (Rutledge, Skandali, Dayan, & Dolan, 2014).

**Additional information collected in the questionnaire at Time Point 2** (which are part of parallel studies conducted in our lab):

Many of the items presented in the survey completed by subjects in Time Point 1 for the parallel studies conducted in the lab were not included At Time Point 2. Questions included at Time Point 2 are: happiness, optimism about getting COVID-19, compliance, emotions, behavioral changes, impact of COVID-19 on income, sense of agency.

In Time Point 2 the following changes were made:

- The question “Do you think COVID-19 presents a real danger to the health of the human population?” was changed to “How likely are people to get COVID-19?” on a scale from 1 (extremely unlikely) to 5 (extremely likely)”. 500 of our participants also completed the original question.
- A question to assess absolute general expectations about getting COVID-19 was added by asking participants “How likely do you think you are to get COVID-19?” on a scale from 1 (extremely unlikely) to 5 (extremely likely)”.

**Attention catch trials**

*Time point 1*

In order to check participants’ engagement and attention, 10 catch trials were inserted in the survey. In the catch trials subjects were asked to select a specific answer (for example: *Please select ‘strongly disagree’*). Specifically, 2 catch trials were present in the intertemporal choice block, 1 in the loss aversion block and one in each of the blocks assessing the following constructs: risk taking, perceived constraints, personal mastery, obsessive-compulsive behaviors, resilience, optimism and apathy. Participants who failed to select the correct answer more than once were excluded from analysis (N=21).

*Time point 2*

2 catch trials were present, specifically in the blocks assessing perceived constraints and personal mastery. Participants who failed to select the correct answer more than once were excluded from analysis (N=0).

**Additional Information on the task**

In the last two blocks of the task (blocks 3-4**)**, participants indicated what they expected the change in portfolio value to be on that trial from -4 (‘decreased a lot’) to +4 (‘increased a lot’) and their confidence in that rating from 1 (‘not confident at all’) to 9 (‘extremely confident’). These ratings occurred directly after they observed the global market on each trial. Each rating had a 8-s time limit. This was followed by the WTP scale and delivery of information or no information as in previous blocks.

**Supplementary Tables**

**Linear Regressions**

**Study 1**

**Time Point 1**

COVID-related information-seeking = β0 + β1* Anxiety Index Score + β2*Age + β3*Gender + β4*Educational level + β5*Income + β6*Political orientation + β7*Ethnicity + β8* Whether they had dependents + β9* satisfaction with their health insurance

| Predictor | t | p |
| --- | --- | --- |
| **Anxiety Index Score** | **7.04** | **< 0.001** |
| Age | 4.74 | < 0.001 |
| Gender | -0.5 | 0.61 |
| Educational level | 2.63 | 0.009 |
| Income | 2.28 | 0.02 |
| Political orientation | -1.57 | 0.11 |
| Ethnicity | 0.94 | 0.34 |
| Dependents | -1.84 | 0.06 |
| Health insurance satisfaction | -0.07 | 0.94 |

Table S1. The table summarizes predictors of linear regression predicting COVID-related information-seeking at Time Point 1. T and p values are reported for each predictor.

COVID-related information-seeking = β0 + β1* Average of the COVID-19-related anxiety questions + β2*Age + β3*Gender + β4*Educational level + β5*Income + β6*Political orientation + β7*Ethnicity + β8* whether they had dependents + β9* satisfaction with their health insurance

| Predictor | t | p |
| --- | --- | --- |
| **Average of the COVID-19-related anxiety questions** | **6.32** | **< 0.001** |
| Age | 4.52 | < 0.001 |
| Gender | -0.70 | 0.48 |
| Educational level | 2.63 | 0.008 |
| Income | 2.15 | 0.03 |
| Political orientation | -1.71 | 0.08 |
| Ethnicity | 1.03 | 0.30 |
| Dependents | -1.89 | 0.058 |
| Health insurance satisfaction | -0.2 | 0.84 |

Table S2. The table summarizes predictors of linear regression predicting COVID-related information-seeking at Time Point 1. T and p values are reported for each predictor.

COVID-related information-seeking = β0 + β1* Short State Anxiety Inventory score + β2*Age + β3*Gender + β4*Educational level + β5*Income + β6*Political orientation + β7*Ethnicity + β8* whether they had dependents + β9* satisfaction with their health insurance

| Predictor | t | p |
| --- | --- | --- |
| **Short State Anxiety Inventory score** | **7.41** | **< 0.001** |
| Age | 4.98 | < 0.001 |
| Gender | -0.20 | 0.84 |
| Educational level | 2.64 | 0.008 |
| Income | 2.58 | 0.01 |
| Political orientation | -1.31 | 0.18 |
| Ethnicity | 0.51 | 0.60 |
| Dependents | -2.09 | 0.03 |
| Health insurance satisfaction | 0.31 | 0.75 |

Table S3. The table summarizes predictors of linear regression predicting COVID-related information-seeking at Time Point 1. T and p values are reported for each predictor.

**Time Point 2**

COVID-related information-seeking = β0 + β1* Anxiety Index Score + β2*Age + β3*Gender + β4*Educational level + β5*Income + β6*Political orientation + β7*Ethnicity + β8* Whether they had dependents + β9* satisfaction with their health insurance

| Predictor | t | p |
| --- | --- | --- |
| **Anxiety Index Score** | **5.26** | **< 0.001** |
| Age | 5.41 | < 0.001 |
| Gender | 1.21 | 0.22 |
| Educational level | 2.12 | 0.03 |
| Income | 1.48 | 0.13 |
| Political orientation | -2.27 | 0.02 |
| Ethnicity | 2.05 | 0.04 |
| Dependents | -1.94 | 0.052 |
| Health insurance satisfaction | 1.96 | 0.05 |

Table S4. The table summarizes predictors of linear regression predicting COVID-related information-seeking at Time Point 2. T and p values are reported for each predictor.

COVID-related information-seeking = β0 + β1* Average of the COVID-19-related anxiety questions + β2*Age + β3*Gender + β4*Educational level + β5*Income + β6*Political orientation + β7*Ethnicity + β8* whether they had dependents + β9* satisfaction with their health insurance

| Predictor | t | p |
| --- | --- | --- |
| **Average of the COVID-19-related anxiety questions** | **4.98** | **< 0.001** |
| Age | 5.33 | < 0.001 |
| Gender | 1.12 | 0.26 |
| Educational level | 2.12 | 0.03 |
| Income | 1.43 | 0.15 |
| Political orientation | -2.35 | 0.01 |
| Ethnicity | 2.09 | 0.03 |
| Dependents | -1.93 | 0.05 |
| Health insurance satisfaction | 1.89 | 0.059 |

Table S5. The table summarizes predictors of linear regression predicting COVID-related information-seeking at Time Point 2. T and p values are reported for each predictor.

COVID-related information-seeking = β0 + β1* Short State Anxiety Inventory score + β2*Age + β3*Gender + β4*Educational level + β5*Income + β6*Political orientation + β7*Ethnicity + β8* whether they had dependents + β9* satisfaction with their health insurance

| Predictor | t | p |
| --- | --- | --- |
| **Short State Anxiety Inventory score** | **4.48** | **< 0.001** |
| Age | 5.36 | < 0.001 |
| Gender | 1.15 | 0.25 |
| Educational level | 2.35 | 0.01 |
| Income | 1.35 | 0.17 |
| Political orientation | -2.30 | 0.02 |
| Ethnicity | 1.91 | 0.056 |
| Dependents | -2.32 | 0.02 |
| Health insurance satisfaction | 1.92 | 0.055 |

Table S6. The table summarizes predictors of linear regression predicting COVID-related information-seeking at Time Point 2. T and p values are reported for each predictor.

**Study 2a**

Willingness to pay = β0 + β1* Trait Anxiety score + β2*Age + β3*Gender

| Predictor | t | p |
| --- | --- | --- |
| Trait Anxiety score | 0.17 | 0.86 |
| Age | -0.20 | 0.84 |
| Gender | -1.09 | 0.28 |

Table S7. The table summarizes predictors of linear regression predicting willingness to pay for information. T and p values are reported for each predictor.

Beta coefficient predicting Willingness to pay from Absolute market change = β0 + β1* Trait Anxiety score + β2*Age + β3*Gender

| Predictor | t | p |
| --- | --- | --- |
| **Trait Anxiety score** | **2.14** | **0.039** |
| Age | -0.61 | 0.54 |
| Gender | 1.11 | 0.27 |

Table S8. The table summarizes predictors of linear regression predicting Beta coefficient predicting Willingness to pay from Absolute market change. T and p values are reported for each predictor.

Beta coefficient predicting Willingness to pay from Signed market change = β0 + β1* Trait Anxiety score + β2*Age + β3*Gender

| Predictor | t | p |
| --- | --- | --- |
| Trait Anxiety score | 0.50 | 0.61 |
| Age | 1.91 | 0.06 |
| Gender | -0.36 | 0.71 |

Table S9. The table summarizes predictors of linear regression predicting Beta coefficient predicting Willingness to pay from Signed market change. T and p values are reported for each predictor.

**Study 2b**

Willingness to pay = β0 + β1*Change in Anxiety score + β2*Group (control/induced anxiety) + β3*Age + β4*Gender

| Predictor | t | p |
| --- | --- | --- |
| Change in Anxiety score | 0.78 | 0.43 |
| Group (control/induced anxiety) | -0.12 | 0.90 |
| Age | -0.41 | 0.68 |
| Gender | 0.23 | 0.81 |

Table S10. The table summarizes predictors of linear regression predicting Willingness to pay for information. T and p values are reported for each predictor.

Beta coefficient predicting Willingness to pay from Absolute market change = β0 + β1* Change in Anxiety score + β2*Group (control/induced anxiety) + β3*Age + β4*Gender

| Predictor | t | p |
| --- | --- | --- |
| **Change in Anxiety score** | **2.15** | **0.037** |
| Group (control/induced anxiety) | -0.79 | 0.43 |
| Age | 0.39 | 0.69 |
| Gender | -0.04 | 0.96 |

Table S11. The table summarizes predictors of linear regression predicting Beta coefficient predicting Willingness to pay from Absolute market change. T and p values are reported for each predictor.

Beta coefficient predicting Willingness to pay from Signed market change = β0 + β1* Change in Anxiety score + β2*Group (control/induced anxiety) + β3*Age + β4*Gender

| Predictor | t | p |
| --- | --- | --- |
| Change in Anxiety score | 1.42 | 0.16 |
| Group (control/induced anxiety) | 0.05 | 0.95 |
| Age | -0.12 | 0.89 |
| Gender | -0.30 | 0.76 |

Table S12. The table summarizes predictors of linear regression predicting Beta coefficient predicting Willingness to pay from Signed market change. T and p values are reported for each predictor.

**Linear Mixed Models**

**Study 2a**

Linear Mixed Model predicting Willingness to Pay.

| Fixed Effects | t | p |
| --- | --- | --- |
| Signed Market Change | 2.25 | 0.02 |
| Absolute Market Change | 3.68 | 0.0006 |
| Trait Anxiety | 0.16 | 0.86 |
| Age | -1.24 | 0.22 |
| Gender | 0.782 | 0.43 |
| Signed Market Change*Trait Anxiety | 0.47 | 0.63 |
| **Absolute Market Change*Trait Anxiety** | **2.15** | **0.03** |

Additionally the following random effects were included: Signed Market Change (grouped by subject), Absolute Market Change (grouped by subject). Using the R package the following code was used: Willingness to Pay ~ Signed Market Change + Absolute Market Change + Trait Anxiety + Age + Gender + Signed Market Change * Trait Anxiety + Absolute Market Change * Trait Anxiety + (Signed Market Change + Absolute Market Change | subject).

Table S13. The table summarizes the fixed and random effects of a Linear Mixed Model predicting Willingness to Pay.

**Study 2b**

Linear Mixed Model predicting Willingness to Pay.

| Fixed Effects | t | p |
| --- | --- | --- |
| Signed Market Change | 2.74 | 0.008 |
| Absolute Market Change | 2.10 | 0.04 |
| Change in Anxiety | 0.36 | 0.71 |
| Age | -1.24 | 0.21 |
| Gender | 0.53 | 0.59 |
| Group | 1.09 | 0.28 |
| Signed Market Change*Change in Anxiety | 2.28 | 0.02 |
| **Absolute Market Change*Change in Anxiety** | **2.22** | **0.03** |

Additionally the following random effects were included: Signed Market Change (grouped by subject), Absolute Market Change (grouped by subject). Using the R package the following code was used: Willingness to Pay ~ Signed Market Change + Absolute Market Change + Change in Anxiety + Group + Age + Gender + Signed Market Change * Change in Anxiety +

Absolute Market Change * Change in Anxiety + (Signed Market Change + Absolute Market Change | subject).

Table S14. The table summarizes the fixed and random effects of a Linear Mixed Model predicting Willingness to Pay.

**Supplementary References**

1. Blain, B., Globig, L. K., & Sharot, T. (2021). Income shock increases delay discounting independently of emotion.
2. Aron, A., Aron, E. N., Tudor, M., & Nelson, G. (1991). Close relationships as including other in the self. Journal of personality and social psychology, 60(2), 241.
3. Blain, B., Globig, L. K., & Sharot, T. (2021). Income shock increases delay discounting independently of emotion. PsyArXiv
4. Davis, M. H. (1983). Empathic concern and the muscular dystrophy telethon: Empathy as a multidimensional construct. Personality and Social Psychology Bulletin, 9(2), 223-229.
5. Engel, C. (2011). Dictator games: A meta study. Experimental economics, 14(4), 583-610.
6. Foa, E. B., Kozak, M. J., Salkovskis, P. M., Coles, M. E., & Amir, N. (1998). The validation of a new obsessive–compulsive disorder scale: The Obsessive–Compulsive Inventory. Psychological Assessment, 10(3), 206.
7. Globig, L.K., Blain, B., & Sharot, T. (2020).When Private Optimism meets Public Despair: Dissociable effects on behavior and well-being. PsyArXiv
8. Kirby, K. N., & Maraković, N. N. (1996). Delay-discounting probabilistic rewards: Rates decrease as amounts increase. Psychonomic bulletin & review, 3(1), 100-104.
9. Kroenke, K., Spitzer, R. L., & Williams, J. B. (2001). The PHQ‐9: validity of a brief depression severity measure. Journal of general internal medicine, 16(9), 606-613.
10. Lachman, M. E., & Weaver, S. L. (1998). The sense of control as a moderator of social class differences in health and well-being. Journal of personality and social psychology, 74(3), 763.
11. Leckelt, M., Wetzel, E., Gerlach, T. M., Ackerman, R. A., Miller, J. D., Chopik, W. J., ... & Back, M. D. (2018). Validation of the Narcissistic Admiration and Rivalry Questionnaire Short Scale (NARQ-S) in convenience and representative samples. Psychological assessment, 30(1), 86.
12. Mann, R. S. (1990). Differential diagnosis and classification of apathy. Am J Psychiatry, 147(1), 22-30.
13. Molina, K. M., Molina, K. M., Goltz, H. H., Kowalkouski, M. A., Hart, S. L., Latini, D., … Gidron, Y. (2013). Revised Life Orientation Test (LOT-R). Encyclopedia of Behavioral Medicine, 1678– 1678.
14. Rutledge, R. B., Skandali, N., Dayan, P., & Dolan, R. J. (2014). A computational and neural model of momentary subjective well-being. Proceedings of the National Academy of Sciences, 111(33), 12252-12257.
15. Smith, B. W., Dalen, J., Wiggins, K., Tooley, E., Christopher, P., & Bernard, J. (2008). The brief resilience scale: assessing the ability to bounce back. International journal of behavioral medicine, 15(3), 194-200.
16. Zhang, D. C., Highhouse, S., & Nye, C. D. (2019). Development and validation of the general risk propensity scale (GRiPS). Journal of Behavioral Decision Making, 32(2), 152-167.
